# Physical-mental health comorbidity: A population-based cross-sectional study

**DOI:** 10.1371/journal.pone.0260464

**Published:** 2021-12-02

**Authors:** Mikk Jürisson, Heti Pisarev, Anneli Uusküla, Katrin Lang, Marje Oona, Lisanna Elm, Ruth Kalda

**Affiliations:** Institute of Family Medicine and Public Health, University of Tartu, Tartu, Estonia; University of Alberta, CANADA

## Abstract

**Background:**

Multimorbidity is associated with physical-mental health comorbidity (PMHC). However, the scope of overlap between physical and mental conditions, associated factors, as well as types of mental illness involved are not well described in Eastern Europe. This study aims to assess the PMHC burden in the Estonian population.

**Methods:**

In this population-based cross-sectional study we obtained health claims data for 55 chronic conditions from the Estonian Health Insurance Fund (EHIF) database, which captures data for all publicly insured individuals (n = 1 240 927 or 94.1% of the total population as of 31 December 2017). We assessed the period-prevalence (3 years) of chronic physical and mental health disorders, as well as associations between them, by age and sex.

**Results:**

Half of the individuals (49.1% (95% CI 49.0–49.3)) had one or more chronic conditions. Mental health disorders (MHD) were present in 8.1% (8.1–8.2) of individuals, being higher among older age groups, women, and individuals with a higher number of physical conditions. PMHC was present in 6.2% (6.1–6.2) of the study population, and 13.1% (13.0–13.2) of the subjects with any chronic physical disorder also presented with at least one MHD. Dominating MHDs among PMHC patients were anxiety and depression. The prevalence of MHD was positively correlated with the number of physical disorders. We observed variation in the type of MHD as the number of physical comorbidities increased. The prevalence of anxiety, depression, and mental and behavioral disorders due to the misuse of alcohol and other psychoactive substances increased as physical comorbidities increased, but the prevalence of schizophrenia and dementia decreased with each additional physical disease. After adjusting for age and sex, this negative association changed the sign to a positive association in the case of dementia and mental and behavioral disorders due to psychoactive substance misuse.

**Conclusions:**

The burden of physical-mental comorbidity in the Estonian population is relatively high. Further research is required to identify clusters of overlapping physical and mental disorders as well as the interactions between these conditions. Public health interventions may include structural changes to health care delivery, such as an increased emphasis on integrated care models that reduce barriers to mental health care.

## Background

Multimorbidity (MM) represents an increasingly prevalent global public health problem. Approximately one in three persons with MM has physical-mental health comorbidity (PMHC) [1]. According to a study by Cassell et al. [2], 9.5% of adult patients from a general practice in England had both physical and mental morbidities, and a recent Canadian study found 8.4% of the adult population had PMHC [3]. The pattern of mental health disorders (MHDs) among MM patients is varied, with depression and anxiety being the most frequent accompanying MHDs [4]. Regarding the types of MHDs involved, a systematic review and meta-analysis studying the prevalence of PMHCs in South Asia established the pooled prevalence of depression as the prevailing MHD among 44% of patients with chronic obstructive pulmonary disease; similar prevalences were found for diabetes (40%), stroke (39%), hypertension (38%), and cancer (37%).

A complex phenomenon, PMHC negatively impacts individuals’ perceptions of lived time and lived space, leading to a loss of agency, heightened uncertainty, and poor well-being [5]. The relationship between mental and physical problems seems to be bidirectional: patients with severe mental health problems such as chronic depression, dementia, or psychotic disorder are at high risk of developing long-term physical conditions, and the risk of mental health problems increases substantially in those with long term physical conditions [1, 6, 7]. The coupling of physical and mental health concerns is a burden not only for patients but for the health care system as well [8], yet the scope of overlapping physical and mental health conditions and associated factors is not well described.

Given that a high proportion of patients with MHDs are managed in a primary care setting, practitioners must be well-versed in identifying MHDs in patients with chronic physical conditions. Although physical and mental health disorders have common risk factors and often co-occur, they are typically treated separately, and physical and mental health care is dichotomized [9]. Reviewing the results from 18 World Mental Health surveys, Scott et al., advised that given the high prevalence and serious clinical consequences of the co-occurrence of PHMCs, attention to their comorbidity should remain a clinical and research priority [10]. A recent scoping review of studies examining the current literature about the prevention, identification, and treatment of physical problems among people with pre-existing MHDs in primary care concluded that the further research is warranted [11]. Given thatEastern European countries are under-represented in the international literature, this population-based study of Estonian citizens using comparable methods will serve as a useful frame of reference for this developing area of research.

This study aims to assess the prevalence of PMHCs in the entire Estonian population, including children, and analyze associations between physical and mental comorbidities.

## Methods

For this population-based cross-sectional study, we obtained data from the Estonian Health Insurance Fund (EHIF), essentially the sole health insurance provider in Estonia covering >94% of the population [12]. We included all subjects from the EHIF database for 2015–2017 and abstracted data for year and month of birth, sex, dates for health claims, type of care (in- and outpatient care, rehabilitation, nursing care, etc), services provided, all diagnosis codes on claims, date of prescription, and the prescription diagnosis code. Study subjects were assigned a unique identifier decoupled from personal identification information to enable longitudinal tracking of care while maintaining patient privacy. The same database and methodology were used when analyzing MM prevalence in Estonia [13].

**Fig 1:**
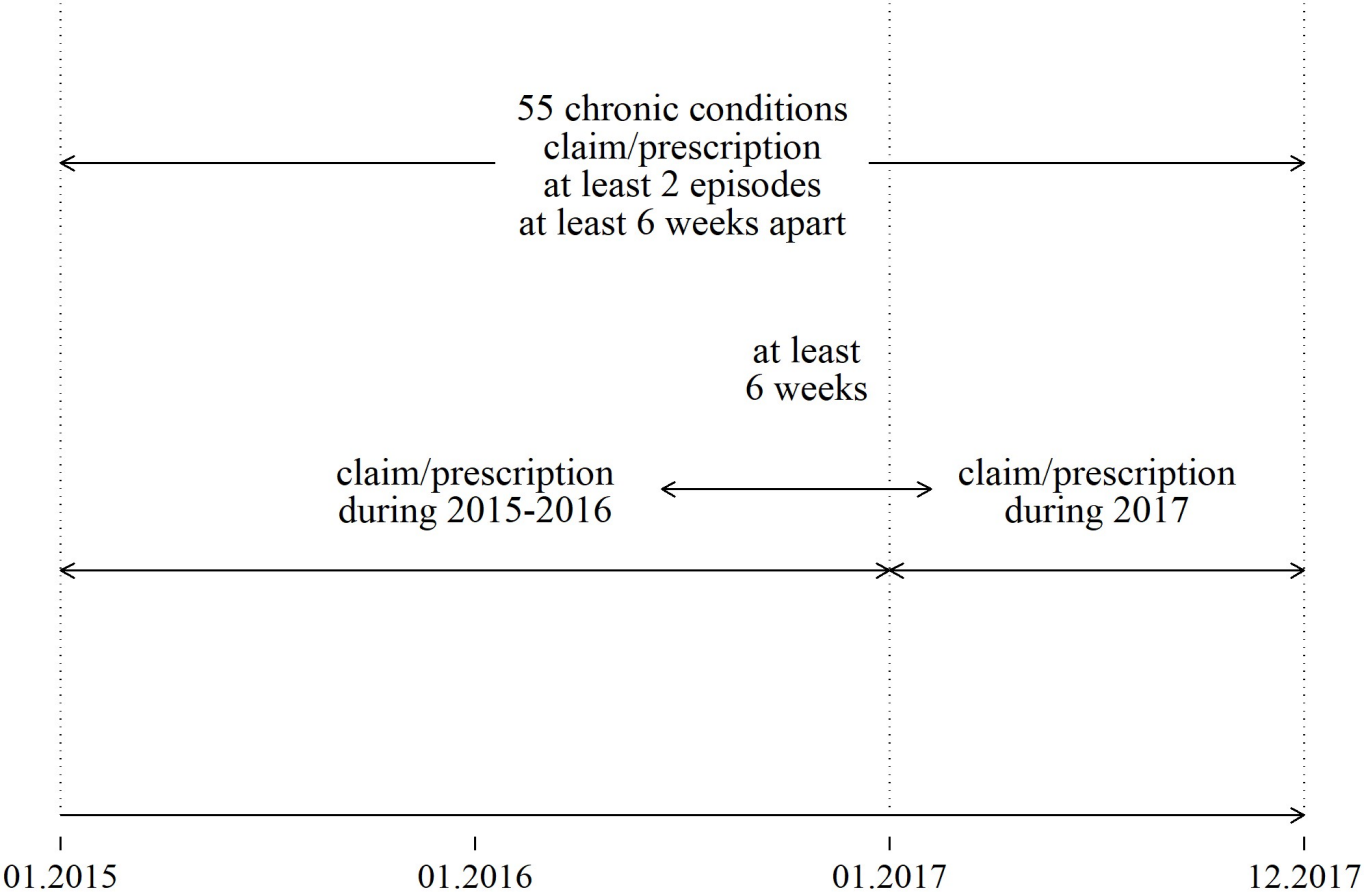
The rationale for a chronic condition case definition.

We selected 55 chronic conditions which we partitioned into physical and mental conditions (S1 Table). The list of conditions was based on previous MM research [1, 14, 15] and adjusted by the authors (MJ, RK, AU, MO, HP) for use in Estonia. As defined by Barnett, et al., we included morbidities that were likely to be chronic: those having a significant impact over at least the most recent year, and with significant impact on patients in terms of the need for chronic treatment, reduced function, reduced quality of life, and risk of future morbidity and mortality [1]. The MHDs analyzed include anxiety, depression, dementia, schizophrenia, disorders due to the use of alcohol, and disorders due to the use of other psychoactive substances. The case definition for a chronic condition was based on the presence of at least two diagnosis codes for that condition from 2015 to 2017: one diagnosis code on a claim or prescription in 2017 plus at least one same or similar code. (Fig 1) To qualify for inclusion, the two identically coded episodes must be from 2015 through 2017 and at least 6 weeks apart. (S1 Table: repetition of diagnostic codes within the boundaries of brackets []) from 2015 to 2017.

This definition enabled us to include chronic conditions that had an impact on a patient at the time of the study, and exclude patients with previously diagnosed but improved conditions, such as those which may have periods of remission (e.g., epilepsy, asthma, pain, or depression). The 6-week interval between the diagnoses reduced over-ascertainment of cases. To identify all patients with chronic physical and mental conditions, the ICD-10 diagnosis codes for main and other (accompanying) diagnoses were used. Inclusion of prescriptions in the analysis enabled the identification of patients whose claims history suggested a single condition whereas the prescription history indicated MM was present. The ascertainment period was extended to 3 years because some people with chronic conditions might visit their physician infrequently. For instance, 83% of publicly insured individuals had only a single visit to a family physician and 63% to a specialist during the 3-year study period [12]. Our analysis end-point was 31 December 2017, at which point we categorized all diseases as being physical or mental disorders and estimated the prevalence of chronic conditions among all publicly insured individuals [1].

The study procedures were conducted following local data protection regulations. The study was approved by the Tartu University Research Ethics Committee.

### Statistical analysis

The outcomes were the prevalence of chronic physical and MHDs (for the general population and population aged 20+ years) and the mean number of those disorders by age and sex. The prevalence and the mean number of MHDs were also reported by the number of physical disorders. To present the variation in the results, we calculated binomial exact 95% confidence intervals (95% CI) for prevalences.

To assess the presence of a correlation between the number of physical disorders and the prevalence of MHDs, the Cuzick test or Chi-square trend test was performed. To evaluate the association between the presence of MHDs with the number of physical disorders, age, and sex, uni- and multivariable Poisson regression was used to estimate prevalence ratios, and 95% CIs. All analyses were performed with STATA version 14.2.

## Results

We analyzed the data of all publicly insured individuals (n = 1 240 927, or 94.1% of the total population) on 31 December 2017 [12, 16] (Table 1). Half of the individuals (49.1% (95% CI 49.0–49.3)) had one or more chronic conditions (the prevalence of all chronic conditions studied is presented in S1 Table). Considering the population aged 20+ years only, the prevalence of at least one chronic condition is 58.4% (58.3–58.6). The presence of MHD was evident in 8.1% (8.1–8.2) of individuals of all ages and 10.1% (10.1–10.2) among those age 20+ years. The prevalence of MHDs was higher among older age groups, women, and individuals with a higher number of physical conditions. The prevalence of MHDs among women was nearly twice as high as in men at prevalence ratio (PR _women/men_) PR _women/men_ = 1.83 (1.80–18.5). Upon adjustment for age and number of physical disorders, this PR_women/men_ decreased to 1.56 (1.54–1.58). The age-specific prevalence of MHDs increased with age but was severely confounded by the number of physical disorders and sex. Upon adjustment for those factors, the PR among ≥85 years decreased from 8.47 (8.17–8.78) to 2.59 (2.49–2.69), suggesting that MHDs in the elderly are primarily associated with a higher number of physical disorders in these age groups and female sex, and not necessarily solely due to older age.

**Table 1:** Prevalence of any chronic disorders, MHDs, and association of MHDs with physical disorders.

		Population (n %)	Prevalence of any chronic disorder (%)	Prevalence of any MHD (% 95% CI)	PR* crude (95% CI)	PR adjusted** (95% CI)
Total		1 240 927 (100.0)	49.1 (49.0–49.3)	8.1 (8.1–8.2)		
Age group (years)	0–24	331 450 (26.7)	18.2 (18.0–18.3)	1.9 (1.8–1.9)	1	1
	25–44	326 460 (26.3)	34.8 (34.6–35.0)	7.7 (7.6–7.8)	4.09 (3.97–4.20)	3.43 (3.34–3.53)
	45–64	323 256 (26.0)	65.6 (65.3–65.8)	11.2 (11.1–11.3)	5.90 (5.74–6.06)	3.18 (3.09–3.27)
	65–84	225 705 (18.2)	85.6 (85.2–85.9)	12.3 (12.2–12.5)	6.52 (6.34–6.70)	2.34 (2.27–2.41)
	≥85	34 056 (2.7)	90.4 (89.4–91.4)	16.0 (15.6–16.5)	8.47 (8.17–8.78)	2.59 (2.49–2.69)
Sex	Men	569 087 (45.9)	43.6 (43.4–43.7)	5.6 (5.6–5.7)	1	1
	Women	671 840 (54.1)	53.8 (53.7–54.0)	10.3 (10.2–10.3)	1.83 (1.80–1.85)	1.56 (1.54–1.58)
Number of physical disorders	0	655 697 (52.8)		3.7 (3.6–3.8)	1	1
	1	234 701 (18.9)		8.4 (8.3–8.5)	2.25 (2.21–2.30)	1.89 (1.85–1.92)
	2	123 991 (10.0)		12.5 (12.3–12.8)	3.36 (3.29–3.42)	2.66 (2.60–2.71)
	3	80 112 (6.5)		15.3 (15.0–15.5)	4.11 (4.02–4.20)	3.25 (3.18–3.33)
	4	54 183 (4.4)		17.3 (17.0–17.6)	4.65 (4.54–4.76)	3.74 (3.64–3.84)
	5	36 225 (2.9)		19.1 (18.7–19.5)	5.13 (5.00–5.27)	4.19 (4.07–4.31)
	6	23 129 (1.9)		20.8 (20.3–21.4)	5.6 (5.43–5.78)	4.62 (4.47–4.78)
	7	14 287 (1.2)		22.2 (21.6–22.9)	5.98 (5.76–6.20)	4.99 (4.80–5.19)
	≥8	18 602 (1.5)		25.9 (25.3–26.6)	6.98 (6.77–7.20)	5.92 (5.72–6.12)

* Prevalence ratio

** Adjusted to all other covariates.

Evidence of PMHC was present in 6.2% (6.1–6.2) of the entire study population () and 7.8% (7.8–7.9) for the population aged 20+ years. Individuals with one physical disease had about twice the prevalence of MHD (8.4 (8.3–8.5)) compared to those without a physical disease (3.7 (3.6–3.8)), and the prevalence of MHDs increased with the number of physical disorders. After adjustment for age and sex, the association between the number of physical disorders and the MHDs decreased from PR _≥8 disorders/0 disorders_ 6.98 (6.77–7.20) to 5.92 (5.72–6.12).

**Fig 2:**
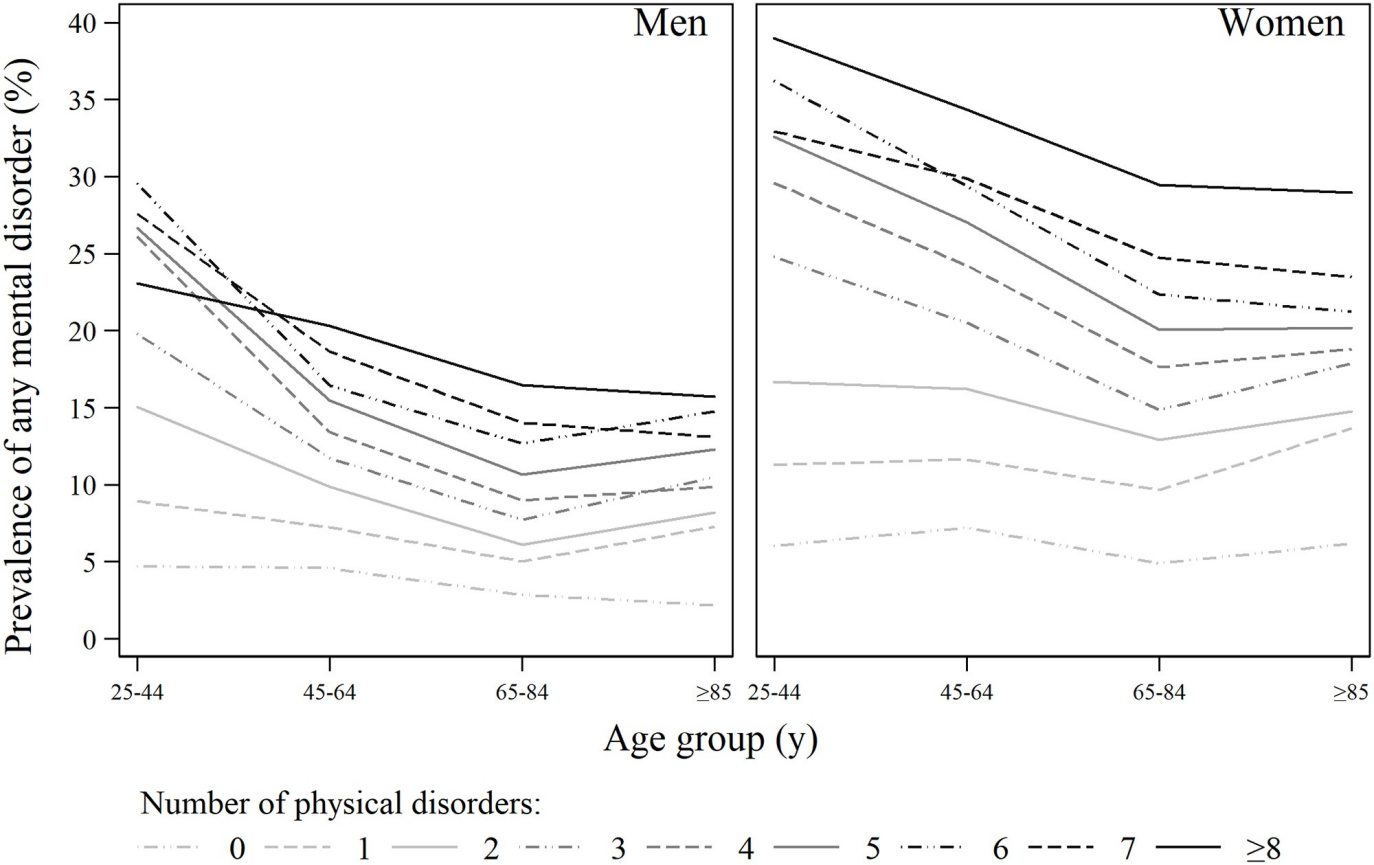
Prevalence of any MHD by the number of physical disorders and age groups in men and women.

The stratified analysis of MHD occurrence by age and number of physical disorders is displayed in Fig 2. Across all age groups, and for both men and women, the prevalence of MHDs was strongly associated with the number of physical disorders, thus confounding the age-specific prevalence of MHDs. Regardless of the number of physical disorders, the prevalence of MHDs was highest among those aged 25–44 years and declined with age in both men and women, except for women with few (1 or 2) physical conditions. The prevalence stabilized or somewhat increased again among those 85 years or older.

Regarding MHDs, the prevalence of anxiety was 4.1%, depression 3.3%, schizophrenia 0.9%, dementia 0.5%, mental and behavioral disorders due to the use of alcohol 0.4%, and other psychoactive substance use 0.2%. The mean number of MHDs in those who had a mental condition and the distribution of these conditions are presented in Table 2. The mean number of any MHD was 1.12 for those who did not have a physical condition. Yet the number of MHDs increased by the number of concomitant physical disorders, ranging from 1.14 among those with only 1 physical condition to 1.17 among those with 8 or more. Although this increase achieved statistical significance due to the large sample size, its clinical significance is likely minor. The type of MHD varied by the number of physical diseases and by groups of MHD. For instance, with an increasing number of physical diseases, we noted a concomitant increase in the prevalence of anxiety, depression, and mental and behavioral disorders due to the misuse of alcohol and other psychoactive substances. In contrast, the prevalence of schizophrenia and dementia decreased with each additional physical disease. After adjusting for age and sex, this negative association changed the sign to become positive again for dementia and mental and behavioral disorders due to psychoactive substance misuse.

**Table 2:** The mean MHDs, distribution and associations between MHDs and the number of physical comorbidities: Crude and adjusted for age and sex.

Number of physical disorders	Mean MHDs	Distribution of MHDs (%, 95% CI)					
		Anxiety	Depression	Dementia	Schizophrenia	Disorders due to use of psychoactive substance	
						Alcohol	Other
0	1.12	45.5 (44.9–46.1)	36.9 (36.3–37.5)	2.3 (2.1–2.5)	19.1 (18.7–19.6)	6.5 (6.2–6.8)	2.3 (2.1–2.5)
1	1.14	48.8 (48.1–49.5)	39.7 (39.0–40.3)	5.0 (4.7–5.3)	12.1 (11.7–12.6)	6.5 (6.1–6.8)	2.1 (1.9–2.3)
2	1.15	50.5 (49.7–51.3)	40.4 (39.6–41.2)	6.9 (6.5–7.3)	9.0 (8.5–9.4)	6.2 (5.8–6.6)	2.5 (2.2–2.7)
3	1.16	51.7 (50.8–52.6)	41.8 (41.0–42.7)	8.3 (7.8–8.8)	6.7 (6.3–7.2)	5.0 (4.6–5.4)	2.0 (1.8–2.3)
4	1.15	52.6 (51.6–53.6)	43.9 (42.9–44.9)	8.1 (7.5–8.6)	5.5 (5.1–6.0)	3.8 (3.4–4.2)	1.5 (1.2–1.7)
5	1.16	53.9 (52.7–55.0)	43.6 (42.4–44.8)	8.7 (8.0–9.4)	4.7 (4.2–5.2)	3.6 (3.2–4.1)	1.6 (1.3–1.9)
6	1.16	54.9 (53.5–56.3)	45.8 (44.4–47.3)	8.1 (7.3–8.9)	3.8 (3.3–4.4)	2.2 (1.8–2.7)	1.1 (0.8–1.5)
7	1.16	56.5 (54.7–58.2)	44.7 (43.0–46.5)	8.0 (7.1–9.0)	3.1 (2.5–3.8)	2.6 (2.1–3.3)	1.4 (1.0–1.8)
≥8	1.17	57.7 (56.3–59.1)	45.3 (43.9–46.7)	7.8 (7.1–8.6)	3.3 (2.8–3.9)	1.8 (1.4–2.2)	0.8 (0.6–1.1)
Association between MHD and the number of physical disorders:							
Crude	1.004^1^ (1.002–1.006)	1.06^2^ (1.06–1.07)	1.05^2^ (1.04–1.06)	1.44^2^ (1.13–1.16)	.73^2^ (.73–.74)	.86^2^ (.85–.87)	.90^2^ (.88–.92)
Adjusted by age and sex	1.01^1^ (1.009–1.015)	1.09^2^ (1.08–1.10)	1.09^2^ (1.08–1.10)	.84^2^ (.83–.85)	.69^2^ (.68–.70)	.92^2^ (.90–.93)	1.18^2^ (1.15–1.21)

^1^ prevalence ratio

^2^ odds ratio.

**Fig 3:**
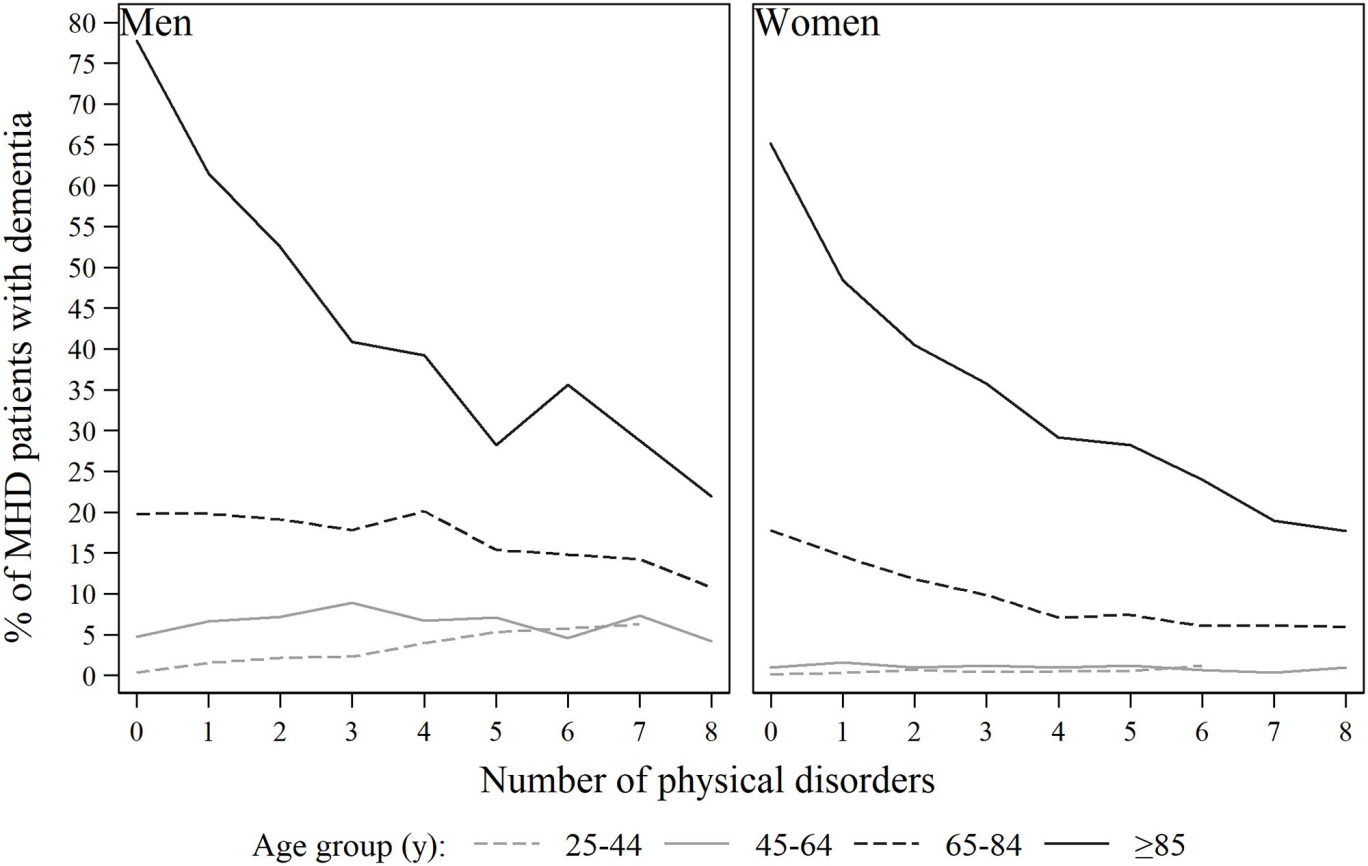
Distribution of patients with dementia by age group and number of physical disorders.

To explore the inverse correlation between the number of physical conditions and dementia, the distribution of dementia by age and number of physical disorders was assessed (Fig 3). The share of dementia among MHDs was considerable in the oldest age groups regardless of the number of accompanying physical disorders.

## Discussion

The analysis revealed that half of the individuals (49.1% (95% CI 49.0–49.3)) had one or more chronic conditions and one in twelve (8.1% (8.1–8.2)) had MHD; PMHC was present in 6.2% (6.1–6.2) of the entire study population. When considering only the adult population aged 20+years, the respective prevalences are 58.4% (58.3–58.6) for one or more chronic conditions and10.1% (10.1–10.2) having an MHD. 7.8% (7.8–7.9) having PMHC in the population aged 20+ years.

Although studies in Eastern Europe are scarce, European researchers investigating the prevalence of physical and mental comorbidity have found similar results. In a recent Scottish study, 8.3% (8.3–8.4) of the sample had PMHC [1], and a recent study by Dai, et al. [3] of over 27 million Canadians aged ≥18 years discovered that 53.9% had one or more chronic diseases, 11.5% had MHD, and 8.4% had PMHC. Cassell et al. [2], studied a random sample of 403 985 adult patients (aged ≥18 years) registered with a general practice in England and found that 9.5% had PMHC. In a study of one US county with 138 000 residents, the PMHC prevalence was 7.9% [4]. The prevalence of all conditions in these two studies is a little higher than in ours because we included the entire insured population and not just those aged ≥18 years.

Similar to other studies [1, 4] the presence of MHDs in chronically ill patients was associated with age and was higher in women than in men. Also, the presence of MHDs increased with the number of physical comorbidities and with age [1, 2]. However, adjusting for the number of physical comorbidities and sex revealed that the high prevalence of MHD in the elderly is primarily associated with a higher number of physical disorders and female sex, and not solely due to increasing age. One of the explanations for the findings could be a survival effect, i.e., only those with few physical diseases tend to survive. Another possible explanation could be the potential difficulty of using validated tools to measure mental health concerns in the elderly due to communication challenges or the overwhelming clinical attention to physical comorbidities.

The most prevalent MHDs accompanying physical conditions were anxiety and depression, and this tendency was further amplified by the number of physical diseases. According to the literature, depression is highly prevalent in patients with physical disorders, particularly in those with severe disorders such as cancer, stroke, and acute coronary syndrome [17, 18]. For example, one in four cancer patients has depression or anxiety [19]. It is difficult to detect whether chronic physical conditions lead to MHDs or vice versa. There is some research showing that these conditions are interrelated: the KAROLA cohort [20], which included coronary heart disease patients, identified anxiety and depression trajectories that were associated with an increased risk of cardiovascular events. Patients with chronic physical illness and concomitant depression or anxiety compared to those with physical illness alone report more symptoms even after controlling for possible confounders [20, 21], therefore, accurate diagnosis of mental disorders among those with a physical illness is essential for optimizing the management of chronic conditions.

Schizophrenia ranked fourth among MHDs accompanying physical conditions, with a finding that it was more prevalent in people with no physical disease. This finding may be explained by its onset at an early age as well as genetic predisposition. Persons with nonschizophrenic psychoses have a higher incidence of somatic diseases compared to people with schizophrenia and nonpsychotic controls [22]. Also, diagnostic overshadowing is an issue: a process by which physical symptoms are misattributed to mental illness and not diagnosed as a medical entity [23]. The combination of schizophrenia with a physical disorder leads to worse outcomes in both conditions [24], and those with schizophrenia show higher mortality compared to the general population [25].

The WHO World Health Survey [26] detected that respondents with depression comorbid with one or more chronic physical diseases had the worst health scores of all the disease states across countries and different demographic characteristics. A systematic review by Katon et al. [27] reported that patients with a chronic physical condition and comorbid depression or anxiety compared to those with a physical condition alone reported significantly more symptoms when controlling for the severity of the disorder. Patients with chronic physical conditions and depression or schizophrenia are also at increased risk of death [24, 28]. The life expectancy of people with mental disabilities is shorter than that of the general population owing to comorbidities and interactions between mental and physical ill-health that are often ignored [29].

The issue of managing MHDs has been recently addressed by WHO, stating that it is essential to scale-up care for people with MHDs and for countries to provide sufficient financial and human resources for mental health care, thus improving access. The WHO implores member states to recognize the cross-cutting nature of mental health issues and the need to integrate mental health services into primary care [30]. Shifting care from institutions to ambulatory primary care settings with improved community-oriented, high-quality services is an important step toward an evidence-based integrated care model [30]. Integrated care is important to improving both physical and mental health outcomes. A systematic literature review concluded that co-occurring physical and mental health disorders can worsen a patient’s course of illness leading to hospital readmission for non-psychiatric reasons [31].

The limitations of the current study may affect generalization. First, a universal definition and list of conditions used for MM research do not exist [32]. For better comparability, we started with the list of conditions found in previous research [1] and added to this list other conditions of interest. Second, it is possible that some people with chronic conditions did not visit a physician or made only one visit over the study period (for example, 83% of publicly insured individuals had a visit to a family physician and 63% to a specialist in 2017 [12]), thus the under-ascertainment of conditions cannot be ruled out. However, we attempted to include only patients with prevalent disorders that impaired the quality of life at the estimation date and excluded those with improved or remitted conditions. We, therefore assumed that people who suffered from a chronic condition visited their physician at least twice within 3 years, at least for a prescription. Third, the EHIF database covers >94% of the population but lacks the data for up to 6% of publicly uninsured individuals [16]. Because all individuals aged 64 years and above are covered by health insurance, we acknowledge a minor under-ascertainment bias among younger age groups, as the health data for the uninsured individuals were not available. Another minor limitation is that not all people who were insured on 31.12.2017 had been continuously enrolled in health insurance coverage, leading to an underestimation of prevalence. Fourth, the validity of EHIF data, established for financial and not health research purposes, does not meet the criteria for use in research. However, this data source has been tested recently [33] and found to be useful for monitoring changes in chronic condition prevalence with a precision sufficient for informing health care policy. Fifth, our cross-sectional design was not able to assess time sequence as physical/mental diseases emerge and is prone to survival bias.

The strength of our analysis lies in the use of a data source with nationwide coverage and complete follow-up. In addition, our definition of morbidity used a three-year ascertainment period such that our study describes people with an active, symptomatic disease that impact wellbeing and quality of life. In other words, our method assumes that a patient who has not received medical attention (i.e., at least two episodes with an identical diagnostic code) within three years is not largely impacted by their condition.

## Conclusions

The burden of PMHC in the Estonian population is high and may be generalizable to other Eastern European countries for which data are currently sparse. This study thus makes an important contribution by quantifying the PMHC burden and analyzing the associations with age and sex. Further research is required to identify clusters of overlapping physical and mental disorders as well as the interactions between these conditions. Public health interventions may include structural changes to health care delivery, such as an increased emphasis on integrated care models that reduce barriers to mental health care.

This study was funded from the core research funding of the Institute of Family Medicine and Public Health, University of Tartu, and the institutional grant IUT34-17.

## Supporting information

S1 Table (DOCX)
