# Supplementary material for: Physical-mental health comorbidity: A population-based cross-sectional study
Source: PLoS One. 2021 Dec 2;16(12):e0260464. doi: 10.1371/journal.pone.0260464 (PMC8638860; doi:10.1371/journal.pone.0260464)
Supplement: S1 Table — (DOCX) [file pone.0260464.s001.docx]

**S1 Table.**

| **Table 1.** List, ICD-10 codes and prevalence of physical and mental disorders in the study | | |  |
| --- | --- | --- | --- |
| **Disorder** | **ICD-10 codes** | **Type** | **Prevalence (%)** |
| Hypertension | [I10–I15] | Physical | 24.49 |
| Painful condition | [G44, R51] [M25.5] [M42–M54] [M77] [M79.1–79.9] [R10.1] [R10.2] [R10.3] [R10.4] [R07.0] [R07.1, R07.2, R07.3, R07.4] [R30] [R52.0] [R52.1] [R52.2] [R52.9] [S22.0] [S22.1] [S12] [S32] [S72] | Physical | 12.37 |
| Rheumatoid arthritis, other inflammatory polyarthropathies, and systemic connective tissue disorders | [M30–M36] [M05–M09, M79.0] [M91] [M15–M19] | Physical | 7.65 |
| Dyspepsia | [K21, K25–K30] | Physical | 7.41 |
| Asthma | [J45–J46] [J30] | Physical | 5.91 |
| Diabetes | [E10–14] | Physical | 5.62 |
| Sleeping disorder | [F51, G47] | Physical | 5.11 |
| Thyroid disorders | [E01–05, E06.1–.9, E07] | Physical | 4.72 |
| Atrial fibrillation | [I44–I45, I47–I49] | Physical | 4.70 |
| Psoriasis or eczema | [L20] [L23] [L28] [L29] [L40] [L50] [L56] | Physical | 4.17 |
| Blindness and low vision | [H17–18, H25–28, H31, H33, H34.1–.9, H35–H36, H43, H47, H54] | Physical | 3.62 |
| Ischaemic heart diseases | [I20–I25] | Physical | 3.44 |
| Heart failure | [I50] | Physical | 3.24 |
| Glaucoma | [H40–H42] | Physical | 3.17 |
| Cancer ** | C00–97, D00–09, D37–48 | Physical | 3.05 |
| Prostate disorders | [N40] [N41] | Physical | 2.52 |
| Disorders of purine and pyrimidine metabolism | [E79, M10] | Physical | 2.07 |
| Anemia | [D50–59, D60–D61, D63–64] | Physical | 1.88 |
| Obesity | [E66] | Physical | 1.64 |
| Female genital noninflammatory disorders of female genital tract | [N81] [N93] [N95] | Physical | 1.57 |
| Neuropathies | [G50–G64] | Physical | 1.56 |
| Disorders of vestibular function | [H81, H82, R42] | Physical | 1.52 |
| Stroke and transient ischaemic attack | [I60–66, I69, G45, I67.2] | Physical | 1.45 |
| Chronic obstructive pulmonary disease/bronchitis | [J40–J44] | Physical | 1.40 |
| Peripheral vascular disease | [I73.0] [I70] | Physical | 0.93 |
| Osteoporosis | [M80, M81, M82] | Physical | 0.89 |
| Epilepsy | [G40–G41] | Physical | 0.84 |
| Hearing loss | [H90–H91] | Physical | 0.74 |
| Migraine | [G43] | Physical | 0.72 |
| Cholelithiasis / Cholecystitis | [K80, K81.1] | Physical | 0.50 |
| Chronic kidney disease | [N18–N19] | Physical | 0.47 |
| Chronic liver disease | [K70–74, K76] | Physical | 0.42 |
| Valve disorders | [I34–I37] | Physical | 0.37 |
| Viral hepatitis | [B18] | Physical | 0.36 |
| Irritable bowel syndrome | [K58] | Physical | 0.33 |
| Parkinson’s disease | [G20, G21, G22] | Physical | 0.31 |
| HIV | [Z21, B20–B24] | Physical | 0.30 |
| Disorders of urinary system | [N39.3, N39.4, R32] | Physical | 0.27 |
| Calculus of kidney and ureter | [N20] | Physical | 0.26 |
| Inflammatory bowel | [K50–K52] | Physical | 0.24 |
| Chronic sinusitis | [J32] | Physical | 0.21 |
| Diverticular disease of intestine | [K57] | Physical | 0.20 |
| Treated constipation | [K59.0] | Physical | 0.16 |
| Multiple sclerosis | [G35] | Physical | 0.12 |
| Coagulation defects | [D65–D69] | Physical | 0.08 |
| Learning disability | [F81] | Physical | 0.06 |
| Anorexia or bulimia | [F50] | Physical | 0.05 |
| Bronchiectasis | [J47] | Physical | 0.05 |
| Celiac disease | [K90.0] | Physical | 0.03 |
| Anxiety and other neurotic, stress-related and somatoform disorders | [F40–F43, F45, F48] | Mental | 4.09 |
| Depression | [F32–F33] | Mental | 3.32 |
| Schizophrenia or bipolar disorder | [F20–F29] [F31] | Mental | 0.85 |
| Dementia | [F00, F01, F02, F03, F05.1, G30, G31, R54] | Mental | 0.48 |
| Mental and behavioral disorders due to use of alcohol | [F10] | Mental | 0.43 |
| Other psychoactive substance misuses | [F11–19] | Mental | 0.16 |
| * [ ] repetition of diagnostic codes within the boundaries of brackets | |  |  |
| ** Every cancer diagnosis observed separately | |  |  |
